# Docosahexaenoic acid-rich algae oil supplementation in mothers of preterm infants is associated with a modification in breast milk oxylipins profile

**DOI:** 10.1186/s12944-023-01870-8

**Published:** 2023-07-14

**Authors:** Hélène Fougère, Karine Greffard, Mireille Guillot, Iwona Rudkowska, Etienne Pronovost, David Simonyan, Isabelle Marc, Jean-François Bilodeau

**Affiliations:** 1 Département de Pédiatrie, CHU de Québec-Université Laval, Québec, QC Canada; 2 Axe Endocrinologie et Néphrologie, CHU de Québec-Université Laval, 2705 Boulevard Laurier, Québec, QC G1V 4G2 Canada; 3 Département de Kinésiologie, Faculté de Médecine, Université Laval, Québec, QC Canada; 4 Plateforme de Recherche Clinique et Évaluative, CHU de Québec-Université Laval, Québec, QC Canada; 5 Département de Médecine, Faculté de Médecine, Université Laval, Québec, QC Canada

**Keywords:** *n-3* fatty acid supplementation, Oxylipins, Breast milk, Prematurity, Inflammation, Bronchopulmonary dysplasia

## Abstract

Oxylipins are derived from enzymatic and non-enzymatic oxidation of *n*-3 and *n*-6 long-chain polyunsaturated fatty acids. They are known to be involved in inflammatory processes. The aim of this study was to describe the breast milk oxylipin profile following a docosahexaenoic acid (DHA) supplementation of mothers of preterm infants. We examined the oxylipins profile in breast milk collected at day 14 post-delivery, of 40 mothers who delivered before 29 weeks of gestation and who were supplemented with either DHA-rich algae oil (S-DHA) or a placebo (PL). These mothers were selected from the MOBYDIck cohort (NCT02371460 registered on 25/05/2015 in ClinicalTrials.gov) according to the supplementation received (S-DHA vs. PL) and the DHA content quartiles as measured in breast milk (Low vs. High) to generate four study groups. Milk oxylipins, as ng/mL of milk, were analyzed by LC-MS/MS. Ten oxylipins derived from DHA were higher in the S-DHA-High group than the other three groups (P < 0.001). The 18-HEPE, was also higher in the S-DHA-High group (0.11 ± 0.01) compared to the other groups (P = 0.0001). Compared to the PL-Low group, there was a reduction in pro-inflammatory prostaglandins found in the S-DHA-High group with lower levels of prostaglandins PGF_2α_ (0.21 ± 0.45 in the S-DHA-High group vs. 1.87 ± 0.44 in the PL-Low group, P = 0.03) and of PGE_2_ (0.33 ± 0.26 in the S-DHA-High group vs. 1.28 ± 0.25 in the PL-Low group, P = 0.04).In sum, the DHA supplementation was linked with a predominance of anti-inflammatory oxylipins in breast milk of mothers who delivered very preterm, like 17(S)-HDHA and 18-HEPE, precursors of D and E resolvins respectively. This was also accompanied with a lower level of pro-inflammatory prostaglandins.

## Introduction

Very preterm infants are more affected by bronchopulmonary dysplasia (BPD), a chronic lung disease, involving an enhanced inflammatory response [1]. With the aim to prevent BPD by attenuating inflammation and oxidative stress, long-chain polyunsaturated fatty acid (LC-PUFA), and in particular docosahexaenoic acid (DHA) at high dose, has been suggested as diet supplementation for preterm infants [2–4]. Although the DINO trial indicated a decrease in BPD among preterm infants following supplementation with a high dose of DHA given to breastfeeding mothers (yielding to approximately 1% total fatty acids in breast milk) [2], two additional randomized clinical trials, including the MOBYDIck trial, suggested that supplementation with high dose of DHA to neonates born very preterm increased BPD compared to a placebo [4, 5]. The underlying mechanisms to explain the increased risk of BPD following DHA supplementation remain to be determined.

Several oxylipins derived from polyunsaturated fatty acids (PUFA) are known to be physiological bioactive lipids with roles in the inflammation process. They are produced by the oxidation of *n*-6 and *n*-3 PUFA via non-enzymatic and enzymatic processes (cytochrome P450, lipoxygenase, cyclooxygenase) [6]. Oxylipins derived from DHA and eicosapentaenoic acid (EPA), known as specialized pro-resolution mediators, participate to the resolution of inflammation. The latter include resolvins, maresins and protectins [7]. Whereas specific arachidonic acid (AA)-derived oxylipins (leukotrienes and prostaglandins) are known to contribute to inflammation processes. We previously characterized the complete breast milk fatty acids (FA) profile ensuing a DHA-rich algae oil supplementation of mothers who gave birth to very preterm infants in a secondary analysis of MOBYDIck trial data [8]. We showed that supplementation with DHA successfully increased the breast milk DHA content but also caused changes on other LC-PUFA like n-6 docosapentaenoic acid (DPA), eicosapentaenoic acid (EPA) and n-3 DPA that are involved in the formation of bioactive lipids. Moreover, the supplementation increased the DHA:AA ratio by 2.9-fold [8]. In the present study, we documented the oxylipins profile derived from LC-PUFA found in breast milk following a high dose of DHA-rich supplementation of lactating mothers that could potentially explain the higher incidence of BPD in the MOBYDIck trial. The hypothesis was to highlight unexpected increase of pro-inflammatory oxylipins altered by LC-PUFA profile following DHA supplementation, which could have influenced the infant immune response, which in turn could have contributed to the development of BPD. More precisely, we have focused the objective on oxylipins profile derived from DHA, EPA and AA in breast milk of mothers who delivered very prematurely (< 29 weeks of pregnancy) and who took supplementation either with DHA-rich algae oil or placebo during the neonatal period.

## Materials and methods

### Study design, settings, and population

This study is a nested mother-infant dyads from a sub-sample of the MOBYDIck trial [5]. The trial was registered with identifier NCT02371460 in to the ClinicalTrials.gov registry on 25/02/2015. Mothers who delivered prematurely from 23^0/7^ to 28^6/7^ weeks of pregnancy and intended to breastfeed their infant were eligible to participate. The mothers were randomized to receive either a mix of corn and soy oils (Placebo group) or DHA-rich algae oil providing 1.2 g/day of DHA (S-DHA group) until the infant reached 36 weeks of postmenstrual age [4]. Major FA in placebo capsules were linoleic acid (LA) at 52%, oleic acid (cis-9 18:1) at 26% and palmitic acid (16:0) at 11%. In capsules from algae *Schizochytrium* sp., major FA were DHA (45%), n-6 DPA (19%) and palmitic acid (17%). The complete capsules FA profile has been published previously [8]. This multicenter study conducted on 461 mothers (n = 232 mothers randomized in the S-DHA group and n = 229 mothers randomized in the Placebo group) was approved by all authorities.

Maternal intake of 1200 mg/day increased breast milk DHA content of approximately 1% of total FA [9]. This increase in breast milk DHA is sufficient to increase DHA levels in infants and are comparable to direct infant supplementation [3]. Protocol for breast milk sampling and FA analysis has been published previously [8].

**Fig. 1:**
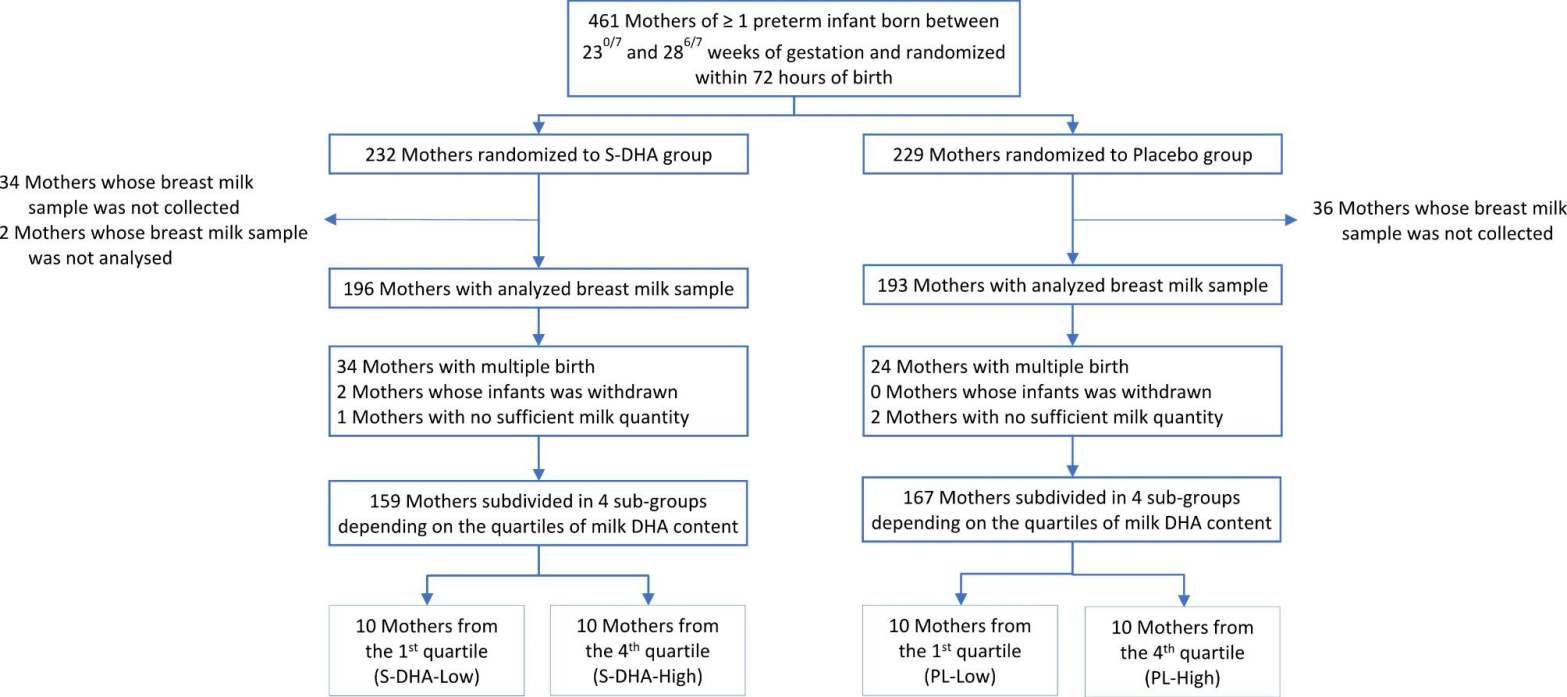
Trial flow diagram according to quartiles of DHA content

For this study, we selected exclusively mothers from a pool of singleton birth with sufficient breast milk sample (n = 159 in the S-DHA group and n = 167 in the Placebo group). Furthermore, we performed a per-protocol analysis of four groups selected according to differing range of milk DHA concentration (Fig. 1). Therefore, ten mothers in the first quartile (low content in DHA) and 10 mothers in the fourth quartile (high content in DHA) were randomly selected in each group (placebo and S-DHA). Thereby, four subgroups were created: low content in milk DHA in the placebo group (PL-Low), high content in milk DHA in the placebo group (PL-High), low content in milk DHA in the S-DHA group (S-DHA-Low) and high content in milk DHA in the S-DHA group (S-DHA-High) with n = 10 per subgroup.

### Breast milk oxylipins analysis

Breast milk samples were slowly thawed by steps to avoid temperature shock and to maintain milk homogeneity. In each extraction tube, 10µL of 25 ng/ml of deuterated standard in ethanol of leukotriene (LT) B_4_-d4, lipoxin (Lx) A_4_-d5, 17(*R*)-resolvin (Rv)D1-d5, RvD2-d5, (5(*RS*)-5-F_2*c*_-IsoP-d11, 5(*R*)-5-F_2*t*_-IsoP-d11, 5(*S*)-5-F_2*t*_-IsoP-d11, LxA_4_-d5, 5(*S*)-HETE-d8, 8-F_2*t*_-IsoP-d4, 15-F_2*t*_-IsoP-d4, 15‑*epi*‑15-F_2*t*_-IsoP-d4, Prostaglandin (PG) E_2_-d9, PGF_2α_-d4, TXB_2_-d4 were added to the 100µL of milk, with 0.6 mL of water, 1.25mL of methanol and vortexed. After a 5-minute centrifugation at 2000 *x g*, supernatants were transferred in a new tube and mixed with 2.5mL of acetic acid (0.176% v/v) before loading them on the solid phase extraction (SPE) cartridge. Conditioning, washing and elution were carried out as previously described [10]. The 8 data points calibration curve was done by the addition of 10µL analytical standard from each stock solution (0.5, 2.5. 5, 12.5, 25, 37.5, 50 and 100 ng/ml) of LTB_4_, LxA_4_, 6(*S*)-LxA_4_, 15(*R*)-LxA_4_, LxB_4_, maresin(Mar) 1, protectin (PD) 1, PDx, RvD1, 17(*R*)-RvD1, RvD2, 17(*R,S*)-RvD4 RvD3, RvD5, RvD5_*n −* 3DPA_, RvE1, PGF_2α_, 5-*trans*-PGF_2α_, 15‑*epi*‑15-F_2*t*_-IsoP, 5‑*epi*‑5-F_2*t*_-IsoP/5-F_2*t*_-IsoP, 15-F_2*t*_-IsoP, 8-F_2*t*_-IsoP, 5‑*epi*‑5-F_2*t*_-IsoP-d11/5-F_2*t*_-IsoP-d11, 5(*R/S*)-5-F_2*c*_-IsoP, 15-F_3*t*_-IsoP, 17-trans-PGF_3α_, PGF_3α_, TXB_2_, 4(*S*)-,7(*S*)-,8(*S*)-,10(*S*)-,11(*S*)-,13(*S*)-,14(*S*)-,16(*S*)- and 20(*S*)-hydroxy docosahexaenoic acid (HDHA), 17(*S*)-HDHA (also known as 17(S)-HDoHE) and (±)-18-hydroxy-5Z,8Z,11Z,14Z,16E-eicosapentaenoic acid (18-HEPE), all acquired from Cayman Chemical (Ann Arbor, MI, USA), to 100µL of a breast milk pool and extracted with the same method as the samples with 10µL of the same deuterated standard (25 ng/ml stock solution) mentioned above. The calibration curve and samples were reconstituted with 75µL of 40% ethanol-0.01% acetic acid and injected with a volume of 50µL. Oxylipins separation gradient was achieved on a high-performance liquid chromatography connected to a mass spectrometry system described elsewhere [10]. Tandem mass spectrometry (MS/MS) was performed using the multiple reaction monitoring mode for quantification. Analyst 1.7 software and SCIEX OS 1.7MQ software (AB Sciex) were used for acquisition and quantification respectively. The limit of detection (LOD) is below 40 pg/ml of breast milk for all analytes and the limit of quantification (LOQ) vary between 40 and 200 pg/ml of breast milk in function of the analyte.

### Statistical analysis

Nonparametric Kruskal-Wallis tests were used to compare continuous data by groups after normality verification; Fisher’s exact tests were used for categorical data comparisons. Linear regression models with generalized estimating equations were performed to estimate mean difference of FA or oxylipins between groups adjusted for neonatal sex, weight and gestational age at birth. All statistics were calculated using SAS Statistical Software v.9.4 (SAS Institute, Cary, NC, USA) with significance level set at P < 0.05 (two-sided), a Tukey-Kramer adjustment for p-values was applied in case of multiple comparisons.

## Results

### Maternal characteristics and breast milk FA composition

Characteristics of the 40 mothers included in this study did not differ between the 4 groups **(**Table 1**)**. The compliance for DHA supplementation was 99 ± 4% in the S-DHA-High group in comparison to 47 ± 38% in the S-DHA-Low group.

**Table 1:** Maternal and neonatal characteristics for breast milk samples analyzed according to the 4 groups

	Mean ± SD, No. (%)				
	**PL-Low**	**PL-High**	**S-DHA-Low**	**S-DHA-High**	***P*** **-value** ^1^
**Mothers**	(n = 10)	(n = 10)	(n = 10)	(n = 10)	
*Pre pregnancy*					
Weight, mean (SD) ^2^, kg	62.78 ± 17.38	69.59 ± 15.32	76.27 ± 12.88	64.53 ± 11.90	0.16
Body Mass Index, mean^2^ (SD), kg/m^2^	23.83 ± 6.70	26.91 ± 5.64	27.75 ± 5.05	23.23 ± 5.00	0.17
*Before delivery*					
Receive corticosteroids, %					
Yes	90.0	100	100	70.0	0.17
No	10.0	0.0	0.0	30.0	
*At delivery*					
Age, mean (SD), y	29.20 ± 5.43	33.10 ± 5.07	31.10 ± 3.84	33.40 ± 4.45	0.22
Delivery Mode, %					
Vaginal	60.0	40.0	50.0	20.0	0.39
Cesarean	40.0	60.0	50.0	80.0	
Parity, mean (SD)	1.70 ± 1.16	1.20 ± 1.23	1.10 ± 1.20	0.80 ± 0.63	0.27
Maternal dietary DHA intake, mean, mg/d	51.9 ± 76.4	196.7 ± 282.1	132.7 ± 212.5	146.7 ± 146.8	0.24
Preeclampsia Eclampsia, %					
Yes	10.0	20.0	10.0	10.0	1.00
No	90.0	80.0	90.0	90.0	
*At 14 days after delivery*					
Compliance, %	71 ± 23	88 ± 21	47 ± 38	99 ± 4	0.002
**Infants**					
Neonatal Sex					
Male	60.0	80.0	80.0	60.0	0.67
Female	40.0	20.0	20.0	40.0	
Gestational age, wk	25.8 ± 1.6	26.6 ± 1.6	27.2 ± 1.0	25.8 ± 1.4	0.12
Birth weight, g	842.8 222.8	896.7 205.4	987.5 260.6	755.9 228.1	0.20

BPD: bronchopulmonary dysplasia, PMA: postmenstrual age, SMOF: SMOFlipid 20% (Fresenius Kabi Canada Ltd, Ontario, Canada)

^1^P-values were estimated by the Mann-Whitney-Wilcoxon test or Fisher?s exact test for categorical variables

^2^For Pl-High and S-DHA-High, n=9

The breast milk FA composition is reported as percentages in Table 2. As expected, and according to our previous publication on its composition [8], the DHA supplementation led to higher levels of DHA, n-6 DPA as well as the ratios DHA:AA and n-3:n-6 within the S-DHA-High group when compared to the other groups. The novelty from the previous study is the stratification in 4 groups according to the DHA levels. The lowest levels in DHA, n-6 DPA, and in the ratios DHA:AA and n-3:n-6 were found in the PL-Low group. We found similar levels of DHA, n-6 DPA, EPA, and the ratios DHA:AA and n-3:n-6 in the PL-High and S-DHA-Low groups. However, as previously reported, the level of DHA in the placebo group was highly related to marine product consumption contrarily to the DHA group [8].

**Table 2:** Fatty acid (FA) composition of maternal breast milk according to the 4 groups

	Mean ± SD				
**Fatty acids**	**PL-Low**	**PL-High**	**S-DHA-Low**	**S-DHA-High**	***P*** **-value** ^1^
	*g/100 g of total fatty acid*				
*n-6* FA					
18:2 n-6 (LA)	13.5 ± 0.90	13.2 ± 0.94	12.1 ± 1.00	13.7 ± 0.91	0.68
18:3 n-6 (ALA)	0.11 ± 0.02	0.07 ± 0.02	0.09 ± 0.02	0.09 ± 0.02	0.56
20:4 n-6 (AA)	0.52 ± 0.04	0.60 ± 0.04	0.48 ± 0.04	0.61 ± 0.04	0.08
22:5 n-6 (n-6 DPA)	0.03 ± 0.02^a^	0.06 ± 0.02^a^	0.08 ± 0.02^a^	0.41 ± 0.02^b^	< 0.0001
*n-3* FA					
18:3 n-3	1.44 ± 0.17	1.61 ± 0.18	1.65 ± 0.19	1.33 ± 0.18	0.63
20:5 n-3 (EPA)	0.04 ± 0.02^a^	0.11 ± 0.02^b^	0.06 ± 0.02^ab^	0.10 ± 0.02^b^	0.01
22:5 n-3 (n-3 DPA)	0.13 ± 0.01^a^	0.22 ± 0.01^b^	0.13 ± 0.01^ac^	0.18 ± 0.01^bc^	< 0.0001
22:6 n-3 (DHA)	0.21 ± 0.05^a^	0.50 ± 0.05^b^	0.39 ± 0.06^ab^	1.46 ± 0.05^c^	< 0.0001
*Ratios*					
DHA:AA	0.40 ± 0.10^a^	0.91 ± 0.11^b^	0.89 ± 0.11^b^	2.40 ± 0.10^c^	< 0.0001
n-3:n-6	0.14 ± 0.01^a^	0.18 ± 0.02^ab^	0.19 ± 0.02^ab^	0.22 ± 0.01^b^	0.01
*Sums*					
∑saturated	45.0 ± 1.88	44.1 ± 1.98	43.8 ± 2.10	42.3 ± 1.90	0.78
∑MUFA	37.2 ± 1.71	38.0 ± 1.80	39.8 ± 1.91	38.2 ± 1.73	0.77
∑PUFA n-6	15.5 ± 0.93	15.0 ± 0.98	13.7 ± 1.04	16.0 ± 0.94	0.45
∑PUFA n-3	2.18 ± 0.21^a^	2.74 ± 0.23^ac^	2.56 ± 0.24^a^	3.41 ± 0.22^bc^	0.002

LA: linoleic acid, AA: arachidonic acid, DPA: docosapentaenoic acid, ALA: alpha-linolenic acid, EPA: eicosapentaenoic acid, DHA: docosahexaenoic acid, MUFA: monounsaturated fatty acid, PUFA: polyunsaturated fatty acid

^1^Estimated using multivariate linear regression models adjusted for neonatal sex, gestational age, birth weight and multiple comparisons with Tukey-Kramer

^a–c^Means within a row with different superscripts are significantly different (P00.05)

### Breast milk oxylipins composition

**Fig. 2:**
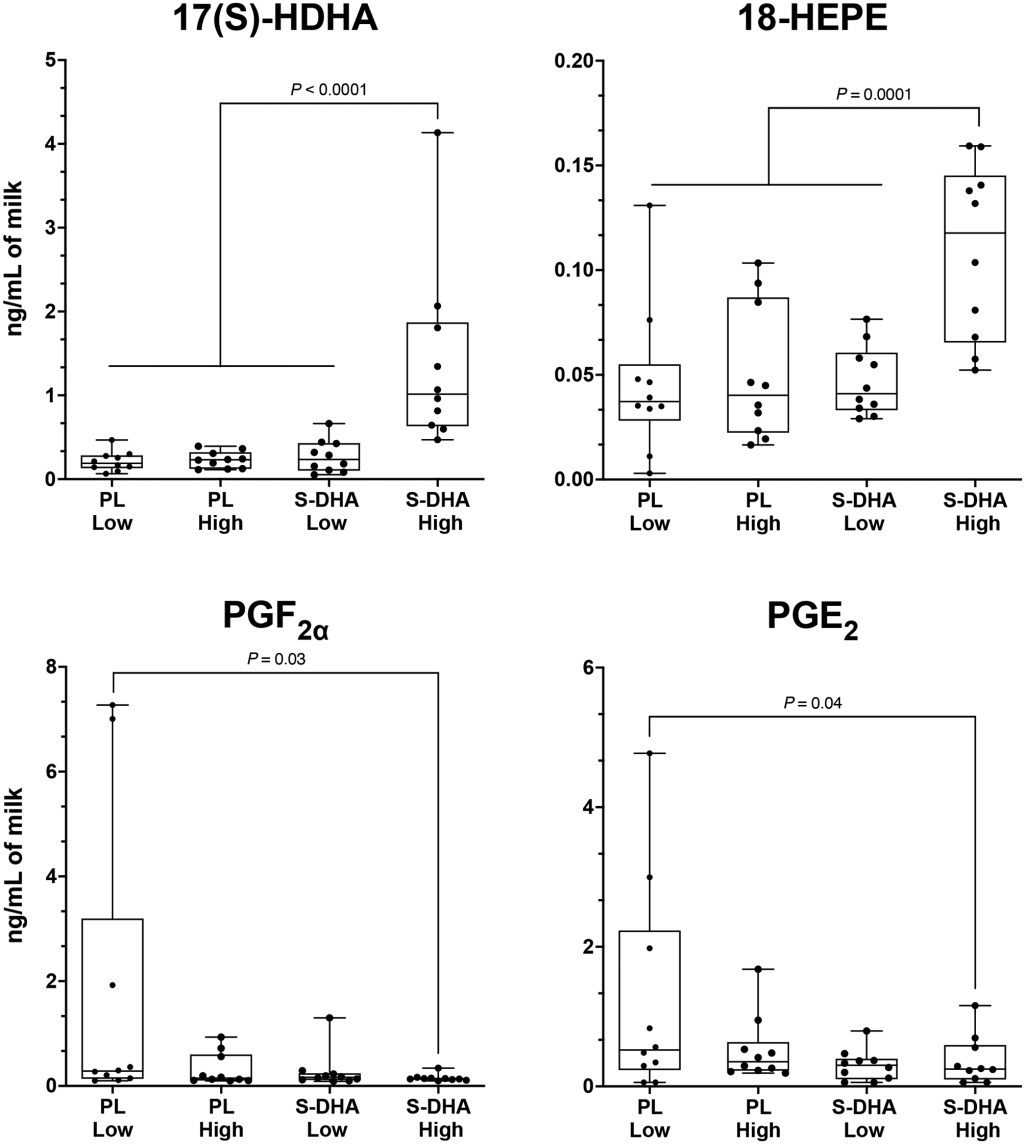
Box plot for 17(S)-HDHA, 18-HEPE, PGE_2_ and PGF_2a_ levels in breast milk in the 4 groups Significant differences (*P*≤0.05) between quartile were observed using multivariate linear regression models adjusted for neonatal sex, gestational age, birth weight followed by multiple comparisons with Tukey-Kramer.

Among the metabolites available, described in the methods section, some were not reported as their levels were too low to be detected in all the breast milk samples. Results were expressed as ng/mL of milk. From 54 FA derivatives targeted for analysis, 17 were quantified in breast milk, and among those, 13 were different between groups (Fig. 2; Table 3). While isoprostanes and lipoxins contents were similar between groups, significant differences were observed in prostaglandin concentrations. As such, the PGF_2α_ was 8.9-fold lower in the S-DHA-High group in comparison to the PL-Low group (P = 0.04). Similarly, a 3.9-fold lower concentration of PGE_2_ was observed in the S-DHA-High group compared to the PL-Low group (P = 0.04). The ten oxylipins derived from DHA were significantly different between the 3 groups (P < 0.0001) with the highest concentration systematically found in the S-DHA-High group while the concentration was similar across the three other groups. Thus, a 6 to 8-fold higher concentration of 17(S)-HDHA was observed in the S-DHA-High group in comparison to all other groups (P < 0.0001). Finally, the 18-HEPE derived from EPA was different between groups (P = 0.0001) with a 2-fold higher concentration in the S-DHA-High group, compared to the three other groups which had similar concentrations (Fig. 2).

**Table 3:** Oxylipins composition of maternal breast milk according to the 4 groups

	Mean ± SD				
Oxylipins	PL-Low	PL-High	S-DHA-Low	S-DHA-High	*P*-value^1^
*ng/mL of milk*					
*Isoprostanes*					
5(*RS*)-5-F_2*c*_-IsoP	0.09 ± 0.02	0.10 ± 0.02	0.12 ± 0.02	0.11 ± 0.02	0.57
5-epi-5-F_2*t*_-IsoP	0.09 ± 0.01	0.09 ± 0.01	0.08 ± 0.01	0.11 ± 0.01	0.76
5-F_2*t*_-IsoP	0.04 ± 0.02	0.06 ± 0.02	0.07 ± 0.02	0.06 ± 0.02	0.57
*Lipoxin*					
15(*R*)-Lipoxin A4	0.03 ± 0.006	0.02 ± 0.007	0.02 ± 0.007	0.04 ± 0.006	0.30
*Prostaglandins*					
PGF_2α_	1.87 ± 0.44^a^	0.31 ± 0.47^ab^	0.22 ± 0.49^ab^	0.21 ± 0.45^b^	0.03
PGE_2_	1.28 ± 0.25^a^	0.52 ± 0.27^ab^	0.31 ± 0.28^ab^	0.33 ± 0.26^b^	0.04
*Oxidation or enzymatic oxidation products of DHA*					
4(*S*)-HDHA	0.10 ± 0.04^a^	0.19 ± 0.04^a^	0.18 ± 0.05^a^	0.59 ± 0.04^b^	< 0.0001
7(*S*)-HDHA	0.08 ± 0.02^a^	0.09 ± 0.03^a^	0.12 ± 0.03^a^	0.38 ± 0.03^b^	< 0.0001
8(*S*)-HDHA	0.10 ± 0.05^a^	0.17 ± 0.049^a^	0.16 ± 0.05^a^	0.68 ± 0.05^b^	< 0.0001
10(S)-HDHA	0.07 ± 0.02^a^	0.07 ± 0.02^a^	0.08 ± 0.02^a^	0.32 ± 0.02^b^	< 0.0001
11(*S*)-HDHA	0.12 ± 0.06^a^	0.12 ± 0.07^a^	0.23 ± 0.07^a^	0.69 ± 0.06^b^	< 0.0001
13(*S*)-HDHA	0.10 ± 0.04^a^	0.10 ± 0.04^a^	0.11 ± 0.04^a^	0.49 ± 0.04^b^	< 0.0001
14(*S*)-HDHA	0.10 ± 0.05^a^	0.13 ± 0.05^a^	0.14 ± 0.05^a^	0.67 ± 0.05^b^	< 0.0001
16(*S*)-HDHA	0.07 ± 0.03^a^	0.10 ± 0.03^a^	0.11 ± 0.04^a^	0.42 ± 0.03^b^	< 0.0001
17(*S*)-HDHA	0.17 ± 0.17^a^	0.19 ± 0.18^a^	0.23 ± 0.19^a^	1.37 ± 0.17^b^	< 0.0001
20(*S*)-HDHA	0.17 ± 0.07^a^	0.23 ± 0.07^a^	0.25 ± 0.07^a^	0.96 ± 0.07^b^	< 0.0001
*Oxidation or enzymatic oxidation products of EPA*					
18-HEPE	0.05 ± 0.01^a^	0.06 ± 0.01^a^	0.05 ± 0.01^a^	0.11 ± 0.01^b^	0.0001
*Sum*					
∑HDHA	1.09 ± 0.44^a^	1.39 ± 0.46^a^	1.60 ± 0.49^a^	6.56 ± 0.44^b^	< 0.0001
∑Isoprostanes	0.22 ± 0.03	0.26 ± 0.04	0.28 ± 0.04	0.27 ± 0.03	0.66
∑Prostaglandins	3.15 ± 0.66^a^	0.83 ± 0.70^ab^	0.53 ± 0.74^b^	0.54 ± 0.67^b^	0.03

^1^ Estimated using multivariate linear regression models adjusted for neonatal sex, gestational age, birth weight and multiple comparisons with Tukey-Kramer.

^a−c^Means within a row with different superscripts are significantly different (P < 0.05).

## Discussion

Significant differences of breast milk DHA content between the 4 subgroups allowed to carry out an accurate study of the effect of DHA while AA level remains relatively unchanged between groups. Various oxylipins derived from milk FA were measured in the current study. Among the metabolites detected, ten were directly derived from DHA (HDHA), while one was a derivative of EPA (18-HEPE) and 6 were derived from AA (3 isoprostanes, 2 prostaglandins and 1 lipoxin). In our study, all the oxylipins derived from DHA were increased in the group with the highest content of DHA in milk (S-DHA-High). More specifically, an increase of the 4(*S*)-HDHA and 10(*S*)-HDHA in breast milk of mothers within the S-DHA-High group was observed. This is in line with a previous study reporting an increase of plasma 4-HDHA and 10-HDHA after maternal supplementation with 800 mg of DHA and 100 mg of EPA during pregnancy [11]. In our study, when the content of DHA in milk was high (S-DHA-High group), the 14(*S*)- and 17(*S*)-HDHA were also the highest. This could indicate an increased anti-inflammatory status since the 17(S)-HDHA is known to be a precursor of D resolvins involved in the resolution of inflammation [12]. Our results are aligned with a previous study which investigated the association between milk FA and their derived-oxylipins in preterm human milk produced during the first month of lactation [13]. They showed a positive association between DHA and the oxylipins 14-HDHA and 17-HDHA. Furthermore, the effect of DHA supplementation on DHA-derived oxylipins is influenced by the dose of DHA received. Indeed, in the current study, the S-DHA-High group had higher concentrations of DHA-derived oxylipins compared to the S-DHA-Low group. Similarly, a study also reported the effect of *n*-3 LC-PUFA supplementation on maternal FA and oxylipins concentrations in blood during pregnancy [14]. The *n*-3 LC-PUFA supplementation in women with higher baseline *n*-3 status was correlated with a higher 7(*S*) and 4(S)-HDHA content in the DHA-supplemented compared to the control, while in women with intermediate or lower baseline *n*-3 status, no differences were found between these groups. Regarding EPA-derived oxylipins favoring the resolution of inflammation [12], the S-DHA-High group had higher concentrations of 18-HEPE, known to be a precursor of E resolvins, compared with other groups, while milk EPA content was similar between PL-High, S-DHA-Low and S-DHA-High groups. However, a positive association between EPA and 18-HEPE in preterm human milk during the first month of lactation has been reported [13].

Regarding the AA-derived oxylipins, known to play a role in the induction of inflammation in several conditions [15], their levels were lower in the S-DHA-High, compared to the PL-Low group. This was observed even though AA remained unchanged between groups. The effects of prostaglandins in breast milk on infants’ health remain to be determined. Therefore, caution in the interpretation of the latter results for prostaglandins is warranted for the time being. However, this is in line with a previous study which showed that in preterm infants, a parenteral lipid emulsion rich in *n*-3 LC-PUFA induced a decrease in blood AA and a decrease of PGF_2α_ [16]. Moreover, in the current study, the level of DHA in milk did not impact the isoprostanes concentration (AA-derived oxylipins) which are accurate markers of oxidative stress [17], showing that the supplementation had apparently no effect on the level of milk oxidation.

Finally, the DHA supplementation had a positive effect on breast milk oxylipins profile with a favorable anti-inflammatory metabolite profile such as higher 17(*S*)-HDHA and 18-HEPE levels without increasing pro-inflammatory eicosanoids. This favorable anti-inflammatory biological profile following PUFA supplementation was previously reported in preterm infants. Indeed, it has been shown that high dose DHA supplementation in mothers of preterm infants inhibit the expression of inflammatory cytokines in breast milk and in the preterm blood cells [18]. Similarly, a study showed that DHA supplementation in lactating mothers who gave birth prematurely reduced pro-inflammatory gene expression in the mammary gland [19]. These favorable inflammatory biological changes following DHA supplementation could have contributed in reducing systemic inflammation in preterm infants, a well-known mechanism implicated in several neonatal morbidities including BPD. Yet, two recent well-designed clinical trials found higher rates of BPD in preterm infants following high dose DHA supplementation in mothers [4, 5]. This unexpected association between a more optimal inflammation profile following DHA supplementation and an increased in the incidence of BPD is concerning and should be further explored. For example, other parameters, such as the sex, may be taken into account. Indeed, DHA supplementation induced an earlier and more important accumulation of oxylipins in human plasma in females than males, but no difference in PUFA precursors, suggesting that the substrate-product relation has a few subtleties [20, 21]. Additionally, it is difficult to differentiate the effect of DHA from that of the variation of DHA:AA ratio. However, even if the supplementation with DHA might have contributed to an imbalance in the n-3 to n-6 ratio that may influence inflammatory reactions, the inflammatory profile is rather favorable and may therefore not be the cause of the increase in BPD. Finally, despite a presumably better inflammatory status, the lack of benefit on BPD observed in the trial [5], could be due to the low capacity of preterm infants to assimilate and metabolize properly these resolvin precursors.

To summarize, the supplementation in DHA-rich algae oil promoted DHA derived anti-inflammatory precursors in breast milk of compliant mothers (S-DHA-High group). The main highlights being the predominance of anti-inflammatory oxylipins in breast milk: the 17(*S*)-HDHA, precursor of D resolvins, and 18-HEPE, precursors of E resolvins. At the same time, we observed a lower level of pro-inflammatory prostaglandins PGF_2α_ and PGE_2_ in the S-DHA-High group compared to the others.

## Limitations of the study

Plasma samples in preterm could have allowed us to determine which metabolites enriched by breast milk are really assimilated by infants. Multiple milk samples taken throughout breastfeeding for all individuals would have confirmed the stability of oxylipins measurements beyond the fourteen days of lactation.

## Conclusion

This study shows that mothers of preterm infants with high DHA content in milk, following a DHA-rich algae oil supplementation, have higher milk oxylipins with anti-inflammatory potential effects derived from LC-PUFA (17(*S*)-HDHA and 18-HEPE) compared to mothers with low dose of DHA in milk. Furthermore, the supplementation in DHA-rich algae oil was associated with a reduction in milk pro-inflammatory eicosanoids (PGE_2_, PGF_2α_). Thereby, the DHA supplementation had a positive effect on breast milk oxylipins profile. These results would need to be validated on the entire cohort. Furthermore, a dose-response relation between DHA supplementation and the oxylipins found in milk could be determined.

## Data Availability

Data cannot be shared. We will examine request from any groups for potential collaboration. and hope to make the data available in the future, provided that we obtain the necessary authorization from the ethics review boards concerned by institutions involved in the trial.
